# C1qtnf6 Expression as a Prognostic Biomarker and Therapeutic Target in Lung Adenocarcinoma: Implications for Immune Infiltration and Tumor Progression

**DOI:** 10.1002/cnr2.70205

**Published:** 2025-06-09

**Authors:** Qincai Li, Hua Zhang, Hai Yun‐Liu, Peifeng Zheng, Xiaogang Xu

**Affiliations:** ^1^ Affiliated Hospital of Jiangxi University of Chinese Medicine Nanchang Jiangxi China; ^2^ College of Chinese Medicine Jiangxi University of Chinese Medicine Nanchang Jiangxi China; ^3^ The Second Affiliated Hospital of Jiangxi University of Chinese Medicine Nanchang Jiangxi China

**Keywords:** C1qtnf6, immune infiltration, LUAD, prognostic marker

## Abstract

**Background:**

The incidence and mortality of lung cancer are increasing every year, making it the primary cause of cancer‐related fatalities globally. Upregulation of C1qtnf6 expression is observed in various human cancers. This study aimed to explore the function of C1qtnf6 in lung adenocarcinoma (LUAD) progression.

**Methods:**

We used The Cancer Genome Atlas (TCGA) dataset to analyze data on lung cancer. The relationship between C1qtnf6 expression and treatment outcomes in patients with LUAD was evaluated using a Kaplan–Meier survival analysis. The receiver operating characteristic (ROC) curve was analyzed to ascertain the diagnostic value of C1qtnf6 in LUAD. Additionally, we performed a correlation analysis to investigate the association between the transcription of *C1qtnf6* and inflammation in LUAD. To create LUAD cell lines with reduced C1qtnf6 expression, *C1qtnf6* was knocked down, and several in vitro analyses were conducted to determine how *C1qtnf6* knockdown affected the proliferation and apoptosis of LUAD cells. Furthermore, the relationship between C1qtnf6 and Interleukin‐10 (IL‐10) was verified. Using the Kyoto Encyclopedia of Genes and Genomes (KEGG) pathway analysis and Gene Ontology (GO) studies, we further examined the impact of *C1qtnf6* knockdown on the biological behavior of LUAD cells.

**Results:**

TCGA dataset analysis revealed that C1qtnf6 expression was much higher in LUAD tissues than in the adjoining normal tissues. Correlation analysis revealed a relationship between C1qtnf6 expression and immune cell infiltration in LUAD. It has been demonstrated that C1qtnf6 expression is closely associated with the tumor immunological milieu, immune checkpoint blockade (ICB), and response to cisplatin treatment. In vitro tests revealed that *C1qtnf6* knockdown reduced IL‐10 levels, accelerated apoptosis, and hindered the growth of LUAD cells, thus indicating a possible link between C1qtnf6 and inflammation.

**Conclusion:**

Our results show that C1qtnf6 may be useful as a prognostic indicator for LUAD.

## 1 Introduction

Lung cancer has become the leading cause of cancer‐related deaths globally, owing to its increasing incidence and mortality rates in recent years [1, 2]. It is classified into two main types: small cell lung cancer (SCLC) and non–small cell lung cancer (NSCLC), with NSCLC accounting for approximately 85% of all lung cancer cases [3, 4]. Lung adenocarcinoma (LUAD) and squamous cell carcinoma are the two basic histological subtypes of NSCLC [5]. LUAD, which is the most frequent of the two, initiates in the lung tissue [6]. It is important to note that a significant number of individuals worldwide succumb to lung cancer each year because of diagnosis at later stages. Hence, gaining insights into the pathogenesis of lung cancer and developing targeted drugs are crucial for its effective treatment [7]. It is critical to diagnose lung cancer as soon as possible, underscoring the significance of early detection [8].

Complement C1q Tumor Necrosis factor‐related protein 6 (*C1qtnf6*) [9], a member of the C1q and tumor necrosis factor (C1QTNF) superfamily, is an inflammation‐related gene [10]. It is overexpressed in several cancers [11]. Research has demonstrated that C1qtnf6 controls apoptosis and angiogenesis in hepatocellular carcinoma via the Akt pathway [12]. C1qtnf6 stimulates proliferation and blocks apoptosis to induce oral squamous cell cancer [13]. However, the role of C1qtnf6 in LUAD remains unclear and requires further investigation.

In this study, we hypothesized that high C1qtnf6 expression in LUAD promotes tumor progression and is associated with poor prognosis, thus being able to potentially serve as a prognostic marker and therapeutic target in LUAD. To test this hypothesis, we systematically analyzed C1qtnf6 expression in LUAD and its relationship with immune infiltration, the tumor microenvironment, and patient survival rate to further explore the clinical value of C1qtnf6 as a prognostic indicator.

Intense research is currently underway to investigate the genetic aspects of lung adenocarcinoma to improve its prognosis, diagnosis, and treatment options [14]. Therefore, the development of a reliable prognostic model is essential for future research on lung cancer incidence [15]. Additionally, the identification of new molecular targets has the potential to advance therapeutic development and improve early detection of gelatinous LUAD. By analyzing the TCGA database, we identified C1qtnf6 as a potential candidate target for lung cancer. C1qtnf6 has been found to be overexpressed in liver cancer and oral squamous cell carcinoma, potentially contributing to tumorigenesis in these tissues. However, the precise function of C1qtnf6 in lung cancer remains unclear. The expression and function of C1qtnf6 in lung cancer were examined by analyzing data from the TCGA database to determine the prognostic importance of this protein in LUAD. The findings of this study would provide valuable evidence for considering C1qtnf6 as a target in the clinical management of lung cancer.

## 2 Methods

### 2.1 Public Data Sources and Processing

LUAD queue patient data according to the legal research target were retrieved from the TCGA database (https://portal.gdc.cancer.gov/) [16]. The TCGA‐LUAD cohort included 599 subjects and contained RNA‐Seq data from 535 LUAD and 59 adjacent normal tissues, including related clinical and *C1qtnf6* transcriptomic data. The data originally downloaded in FPKM format were converted to TPM and log2 formats for further study. The cases chosen for analysis comprised gene expression data for *C1qtnf6* and associated clinical information, including age, sex, smoking status, T, N, and M stages, pathological stage, tumor location, and survival status. The R software v3.6.3 was used to conduct all analyses. To create expression analysis plots and Kaplan–Meier (KM) survival curves, we specifically employed the following three R packages: “ggplot2,” “survminer,” and “Survival.” To assess significance, we used the log‐rank test and calculated the *p*‐value and hazard ratios (HR) with a 95% confidence interval (CI) using univariate Cox proportional hazard regression.

### 2.2 LinkedOmics Database

In this study, we used the data for *C1qtnf6* expression in LUAD from the LinkedOmics database (http://www.linkedomics.org/login.php) [17]. We performed the Kyoto Encyclopedia of Genes and Genomes (KEGG) and gene ontology (GO) enrichment analyses for C1qtnf6 signaling in LUAD and visualized the data using the R package “ggplot2.”

### 2.3 Single‐Sample Gene Set Enrichment Analysis (**ssGSEA)**

To ascertain the relative tumor invasion levels of immune cell types, ssGSEA was carried out using the R “GSVA” package in conjunction with gene expression levels found in the published featured genes list [18].

### 2.4 Cell Culture

H1975 and A549 cell lines were obtained from the American Type Culture Collection (Manassas, VA, USA). H1975 cells, harboring an EGFR mutation, are commonly used to study EGFR‐related signaling pathways and drug sensitivity in lung cancer. A549 cells, containing a KRAS mutation, are widely employed to investigate proliferation, apoptosis, and resistance mechanisms in LUAD. Therefore, these two cell lines provide a comprehensive model for studying the role of C1qtnf6 in different cellular environments of LUAD. The cells were cultured in RPMI 1640 medium containing 10% fetal bovine serum, 100 U/mL penicillin, and 100 U/mL streptomycin, in a humidified incubator at 37°C with 5% CO_2_ [19].

### 2.5 Transfection of siRNA

According to the standard protocol, H1975 and A549 cells were seeded in six‐well plates, and 40%–60% confluence was achieved. A small interfering RNA (siRNA) targeting human *C1qtnf6* was synthesized by RiboBio (Guangzhou, China). The cells were transfected in Opti‐MEM reduced‐serum medium (Gibco, USA) using Lipofectamine 3000 reagent (Invitrogen, USA). After 48 h, the cells were extracted and a quantitative reverse transcription polymerase chain reaction (RT‐qPCR) was performed to assess the transfection effectiveness.

### 2.6 MTS Determination

The MTS colorimetric assay was used to assess cell viability and proliferation. *C1qtnf6*‐knockdown H1975 and A549 cells (2000 cells/well) were seeded onto 96‐well plates along with control cells. Each well contained 100 μL of fresh medium, which was replenished every 24 h. At 24, 48, 72, and 96 h, 10 μL of MTS solution was added to each well. After a 30‐min incubation, the absorbance of each group was measured at 490 nm using an enzyme label reader. Readings were used for subsequent statistical analyses [20].

### 2.7 ELISA Detection

The IL‐10 ELISA kit was used as directed (Solarbio, Catalog No: SEKH‐0018). Cells (2 × 10^5^) were seeded on six‐well plates and cultured for a full day. Following cell adhesion, serum‐free DMEM was added, and the medium was adjusted. After collecting the culture media, the cytokine content was measured 24 h later at 490 nm.

### 2.8 Flow Cytometry

After two washes with phosphate‐buffered saline, cells were centrifuged to extract the supernatant. After resuspension at room temperature, they were gently mixed with 5 μL of Annexin V‐FITC and 195 μL of Annexin V‐FITC binding solution. Next, 10 μL of propidium iodide stain solution was added to each tube, gently mixed, and incubated for 30 min at room temperature. Apoptosis was detected by CytoFLEX flow cytometry for subsequent analysis and presentation [21].

### 2.9 Statistical Analysis

C1qtnf6 expression in lung cancer and normal tissues was compared using Student's t‐test. Single‐factor Cox regression analysis was used to obtain HR and *p*‐values. Kaplan–Meier analysis was employed to stratify survival time based on high or low expression of C1qtnf6. *p* < 0.05 was regarded as the significance level.

## 3 Results

### 3.1 Elevated Expression of C1qtnf6 Based on Pan‐Cancer Analysis

**FIGURE 1:**
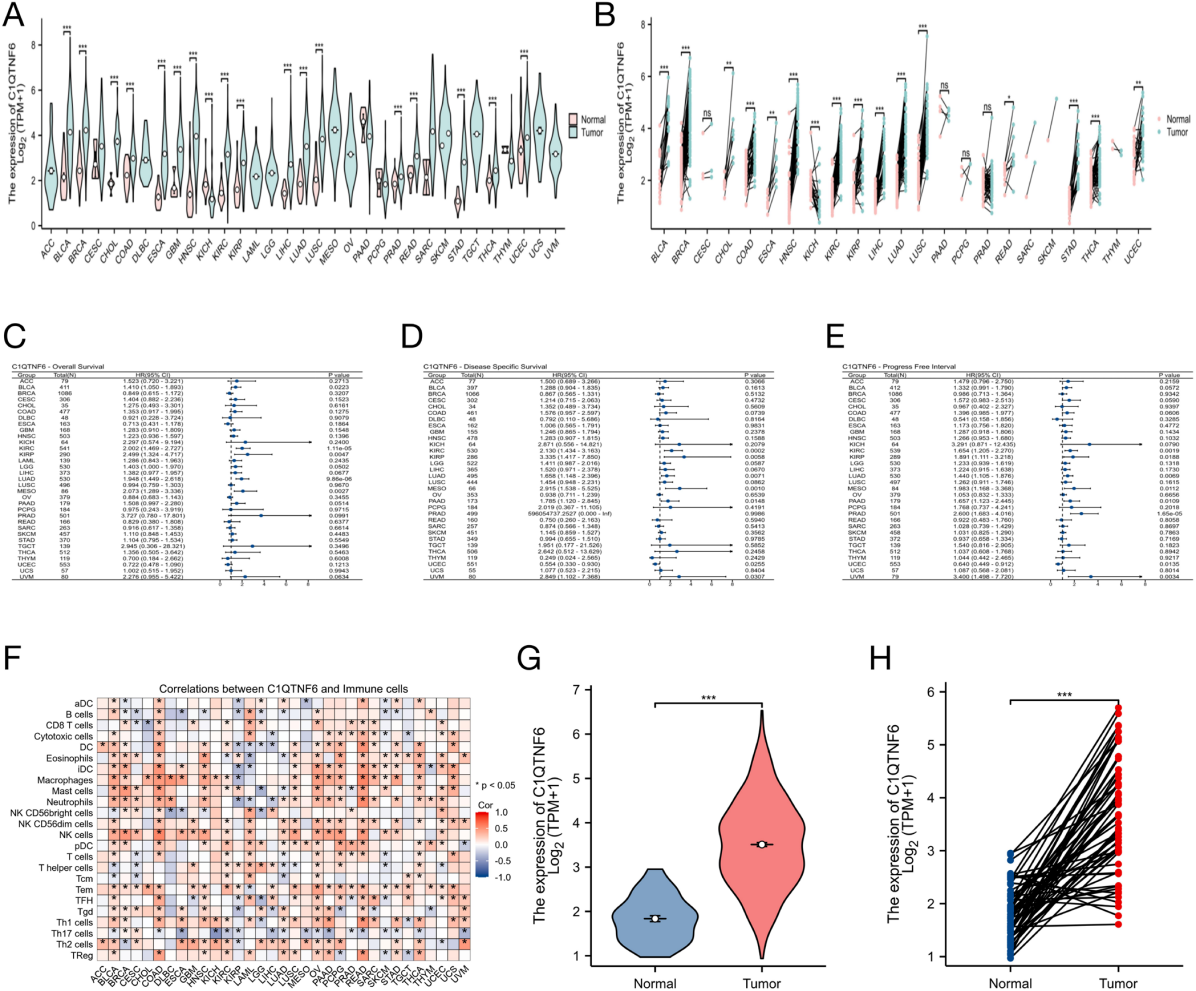
Differing C1qtnf6 expression in tumors. (A) GTEx database and TCGA database revealed C1qtnf6 expression in normal and tumor tissues. (B) According to TCGA data, C1qtnf6 levels in the tumor and corresponding normal tissue vary in different cancers. (C–E) Cox regression was used to analyze 33 cancer outcomes, including OS, DSS, and PFI. (F) Relationship between C1qtnf6 and immune cells in different tumors. mRNA expression of *C1qtnf6* in LUAD based on TCGA data. (G) *C1qtnf6* mRNA expression in patients with LUAD. (H) C1QTNF6 protein expression in patients with LUAD. *p < 0.05, **p < 0.01, ***p < 0.001.

Pan‐cancer analyses using TCGA data showed that C1qtnf6 was significantly upregulated in many tumors, including BLCA, BRCA, STAD, and LUAD, compared with the corresponding normal tissues (Figure 1A). When examining C1qtnf6 expression across 23 types of tumors and their matched normal tissues, the results were largely consistent with those of the unmatched samples, confirming the widespread upregulation of C1qtnf6 in cancer tissues (Figure 1B). To assess the clinical significance of C1qtnf6 expression, we analyzed its association with patient outcomes across a pan‐cancer dataset using Cox regression analysis of 33 cancer types, evaluating survival measures, including overall survival (OS), disease‐specific survival (DSS), and progression‐free survival (PFS) (Figure 1C–E). These results indicate that higher C1qtnf6 expression is often associated with poorer survival outcomes across multiple cancer types, emphasizing its prognostic relevance.

Furthermore, we investigated the correlation between C1qtnf6 expression and immune cell infiltration across different tumor types, which revealed notable associations with specific immune cell types, suggesting a potential role for C1qtnf6 in modulating the tumor immune microenvironment (Figure 1F).

### 3.2 C1qtnf6 Expression in LUAD Patients

The mRNA and protein expression levels of C1qtnf6 in LUAD were determined by analyzing expression data from TCGA and HPA. Additionally, unpaired data analysis revealed that the expression of *C1qtnf6* mRNA in LUAD tissues (*n* = 535) was much higher than that in the adjacent normal tissues (*n* = 59) (*p* < 0.001) (Figure 1G). The expression of *C1qtnf6* mRNA in LUAD tissues (*n* = 57) was considerably higher than that in neighboring normal tissues (*n* = 57) according to paired data analysis (Figure 1H, *p* < 0.001).

### 3.3 Association Between Clinical Characteristics of Patients With LUAD and C1qtnf6 Expression

**FIGURE 2:**
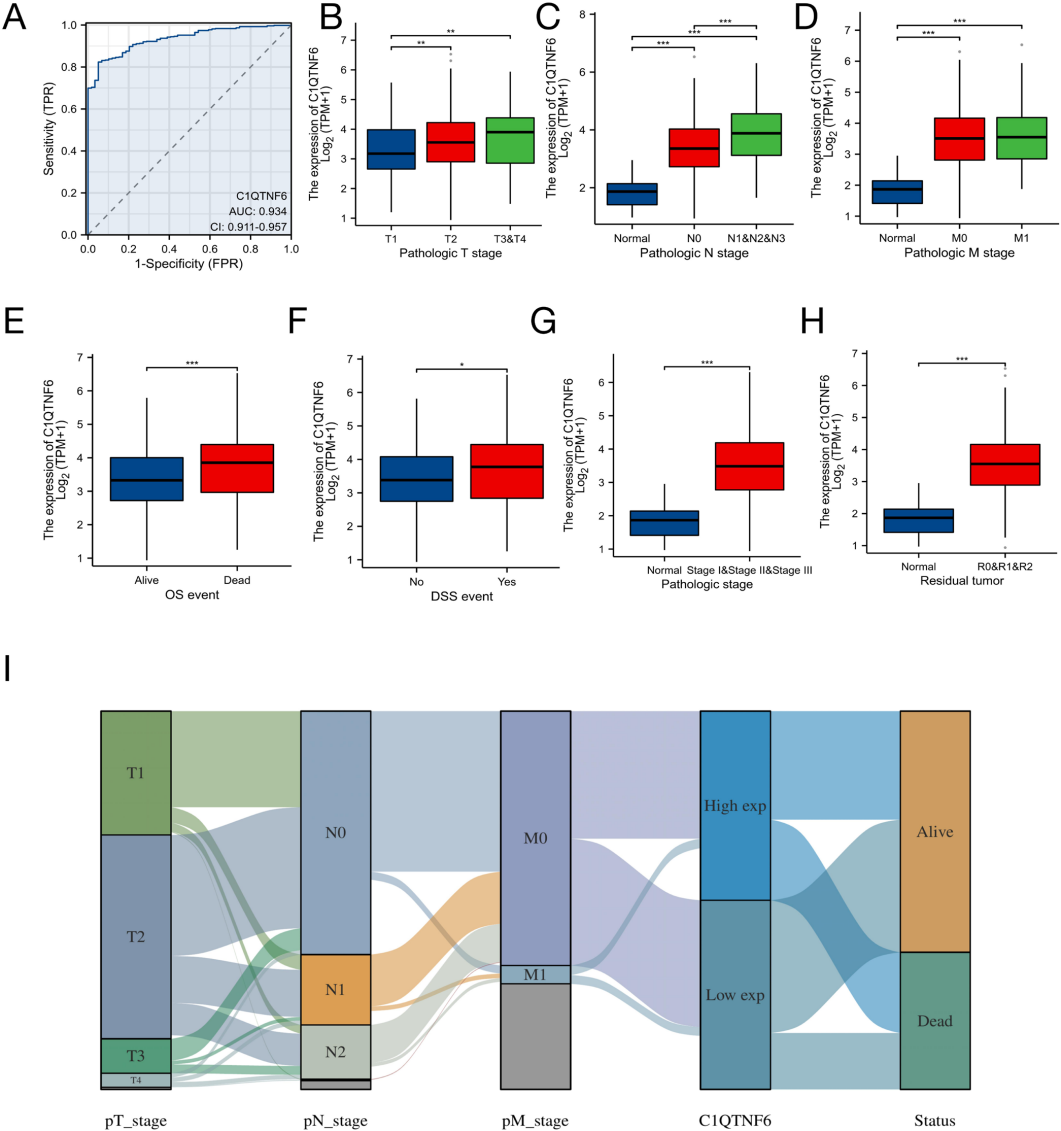
Correlation analysis of C1qtnf6 expression and clinical characteristics. (A) C1qtnf6 receiver operating characteristic (ROC) analysis in LUAD. (B) C1qtnf6 expression and T stage. (C) Relationship between C1qtnf6 expression and N stage. (D) Relationship between C1qtnf6 expression and M stage. (E) Relationship between C1qtnf6 expression and OS. (F) Relationship between C1qtnf6 expression and DSS. (G) Relationship between C1qtnf6 expression and pathologic stage. (H) Relationship between C1qtnf6 expression and residual tumor. (I) Analysis of association between clinical features and C1qtnf6 expression. *p < 0.05, **p < 0.01, ***p < 0.001.

Using logistic regression analysis, C1qtnf6 expression was found to be a categorically dependent variable linked to poor prognostic clinical characteristics (Table 1). To assess the diagnostic utility of C1qtnf6, we performed receiver operating curve (ROC) analysis of the gene expression data (Figure 2A). Area under the curve (AUC) was found to be 0.934 (CI = 0.911–0.957). These findings suggest that the expression of C1QTNF6 is useful for the diagnosis of LUAD. As shown in Figure 2B–H, there was a significant correlation between high C1qtnf6 expression and T stage (*p* < 0.01), N stage (*p* < 0.001), M stage (*p* < 0.001), OS (*p* < 0.001), DSS (*p* < 0.05), pathological stage (*p* < 0.001), and residual tumors (*p* < 0.001). These findings offer a new approach to the early diagnosis of LUAD.

**TABLE 1:** Association between C1qtnf6 expression and clinical prognostic characteristics by logistic regression analysis.

Characteristics	Total (*N*)	OR (95% CI)	*p*
Pathologic T stage (T2 & T3 & T4 vs. T1)	536	1.787 (1.240–2.577)	**< 0.01**
Pathologic N stage (N1 & N2 & N3 vs. N0)	523	1.879 (1.296–2.723)	**< 0.001**
Pathologic M stage (M1 vs. M0)	390	1.054 (0.468–2.372)	> 0.05

*Note:* Bold indicates significant values.

**FIGURE 3:**
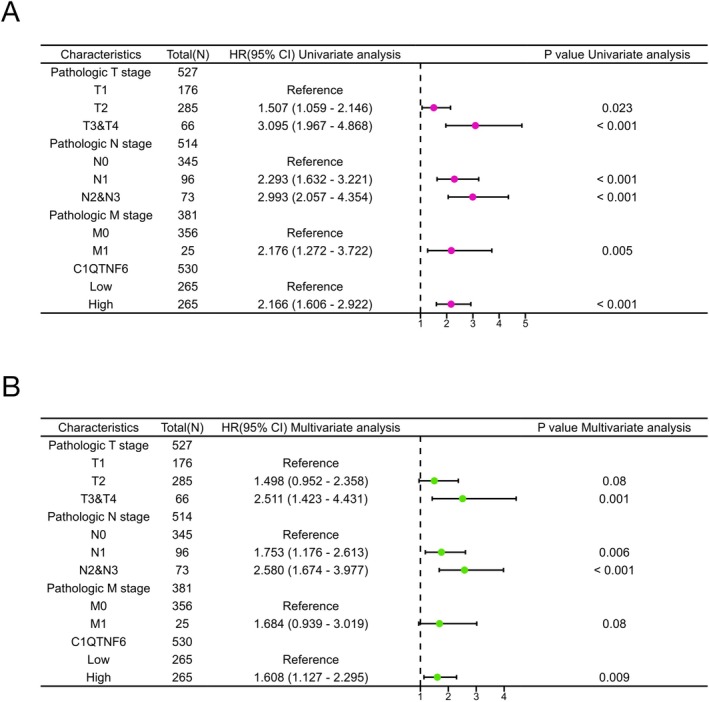
Univariate and multivariate Cox regression analyses of C1qtnf6 expression and clinical characteristics on the overall survival (OS) of patients with LUAD. (A) Univariate analysis demonstrated a link between C1qtnf6 expression, T stage, N stage, M stage, and OS. (B) Multivariate analysis revealed that *C1qtnf6* mRNA expression, T stage, N stage, and M stage were all independent predictors of LUAD OS. *p < 0.05, **p < 0.01, ***p < 0.001.

Based on the median expression value of C1qtnf6 expression, 265 patients were classified as having high expression, and the same number of patients were classified as having low expression to examine the association between C1qtnf6 expression and OS. C1qtnf6, T stage, N stage, and M stage were identified as high‐risk variables by univariate analysis of TCGA data (Figure 3A). C1qtnf6 was shown to be an independent prognostic factor for OS in LUAD through multivariate analysis (HR = 1.608, 95% CI = 1.127–2.295; *p* = 0.009) (Figure 3B).

### 3.4 High C1qtnf6 Expression as an Independent Risk Factor for OS

**FIGURE 4:**
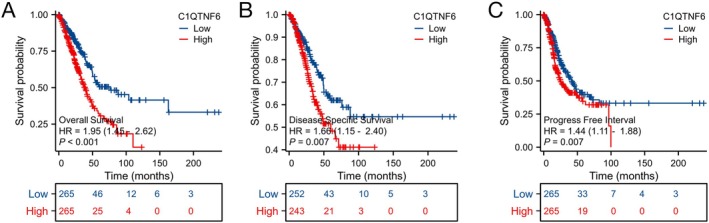
Relationship between C1qtnf6 expression and LUAD prognosis. (A) TCGA‐based overall survival, (B) disease‐specific survival, and (C) progression‐free survival.

**FIGURE 5:**
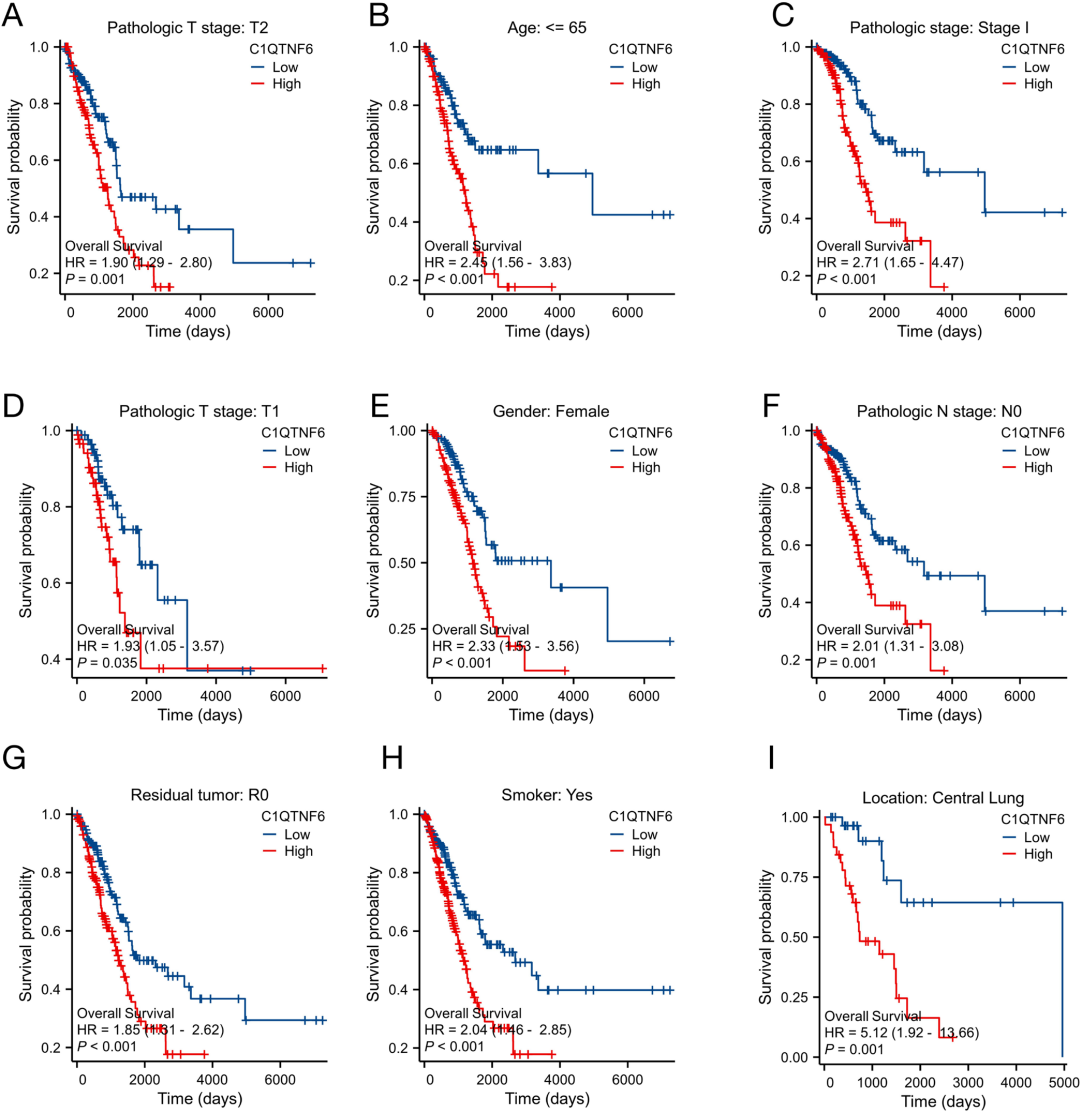
Subgroup analysis and Kaplan–Meier curve for C1qtnf6 expression. (A) T2 stage. (B) Age ≤ 65 years. (C) Stage I. (D) T1. (E) Female sex. (F) N0. (G) R0. (H) Smoker. (I) Central lung location.

Individuals with higher levels of C1qtnf6 expression had a worse prognosis than those with lower levels of C1qtnf6 expression (HR = 1.95; 95% CI: 1.45–2.62, *p* < 0.001, Figure 4A), according to the Kaplan–Meier survival analysis of the TCGA‐LUAD dataset. The cohort sizes in Figures 3 and 4 varied owing to the availability of specific clinical data for T, N, and M stages, and C1qtnf6 expression in the TCGA dataset. Not all patients had complete data for each clinical parameter, leading to differences in the cohort sizes for each analysis. Specifically, Figure 3 includes cohort sizes of 527 (T stage), 514 (N stage), and 381 (M stage), while Figure 4 shows cohort sizes of 495 and 530, based on data availability. In the LUAD dataset, we examined Kaplan–Meier plots for platinum‐free interval (PFI) and DSS. Shorter DSS (HR = 1.66; 95% CI: 1.15–2.40, *p* = 0.007, Figure 4B) and PFI (HR = 1.44; 95% CI: 1.11–1.88, *p* = 0.007, Figure 4C) were linked to low C1qtnf6 expression. The PFI serves as a critical prognostic marker in LUAD, reflecting the time from the end of platinum‐based chemotherapy to cancer recurrence. A longer PFI is generally associated with better outcomes as it indicates prolonged disease control following chemotherapy. For patients who receive epidermal growth factor receptor–tyrosine kinase inhibitor (EGFR‐TKI) therapy after relapse, the PFI remains relevant as long as no additional platinum‐based therapies are introduced. This calculation helps assess the effectiveness of initial treatment and guide subsequent therapeutic strategies. High expression of C1qtnf6 is linked with T1 and T2 stages (*p* = 0.001), age < 65 years (*p* < 0.001), Stage I (*p* < 0.001), and T1 stage (*p* = 0.035), female sex (*p* < 0.001), N0 stage (*p* = 0.001), R0 (*p* < 0.001), smokers (*p* < 0.001), and central lung location (*p* = 0.001), according to an analysis of various clinical characteristics. The central lung location (*p* = 0.001) and other characteristics of patients with LUAD as described above were significantly associated with a poor prognosis (Figure 5A–I).

### 3.5 Creation and Assessment of a Prognostic Model for Individuals With LUAD

**FIGURE 6:**
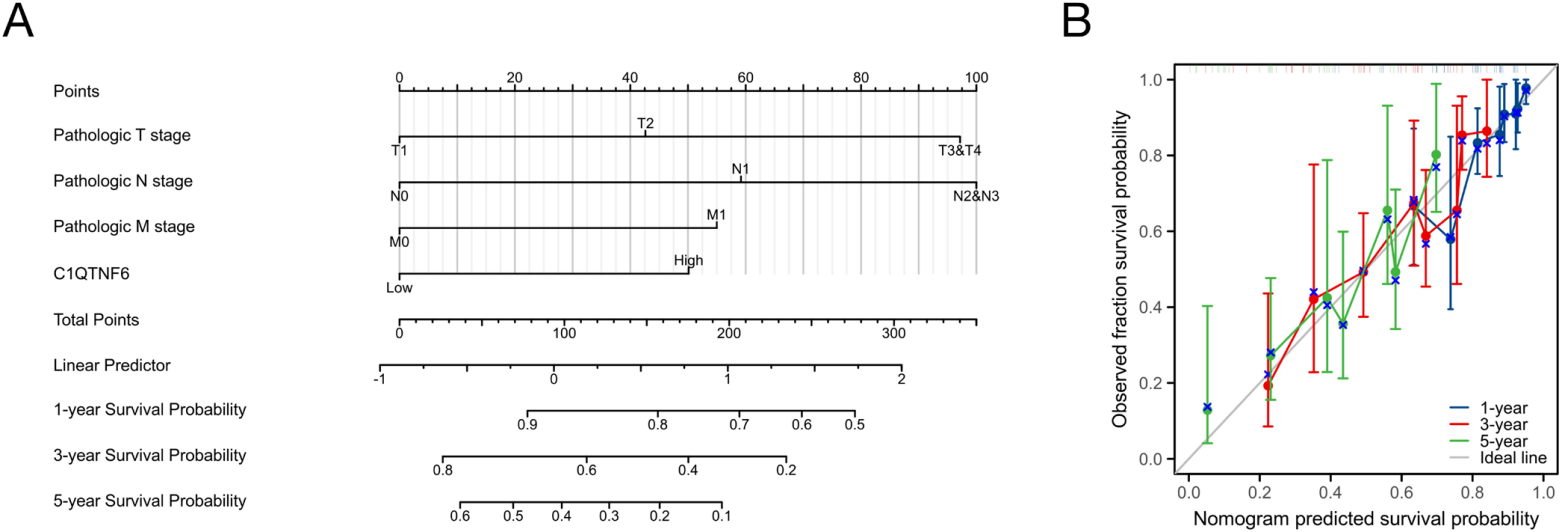
(A) Integration of 1‐, 3‐, and 5‐year survival for C1qtnf6 expression and independent prognostic variables for LUAD obtained from analyzing the TCGA database into a nomogram. (B) Column chart calibration curve prediction.

Univariate and multivariate regression analyses indicated that the only predictive factor for LUAD was C1qtnf6. C1qtnf6 expression levels, TNM stage, age, sex, and pathological stage were combined to create a nomogram that was used to estimate 1‐, 3‐, and 5‐year survival probabilities (Figure 6A). Calibration curves validated the feasibility of this method (Figure 6B). In summary, there was a strong correlation between C1qtnf6 expression and the OS of patients with LUAD, implying that it may be employed as a prospective predictor of survival in LUAD patients.

### 3.6 Relationship Between Immune Infiltration and C1qtnf6 Expression

**FIGURE 7:**
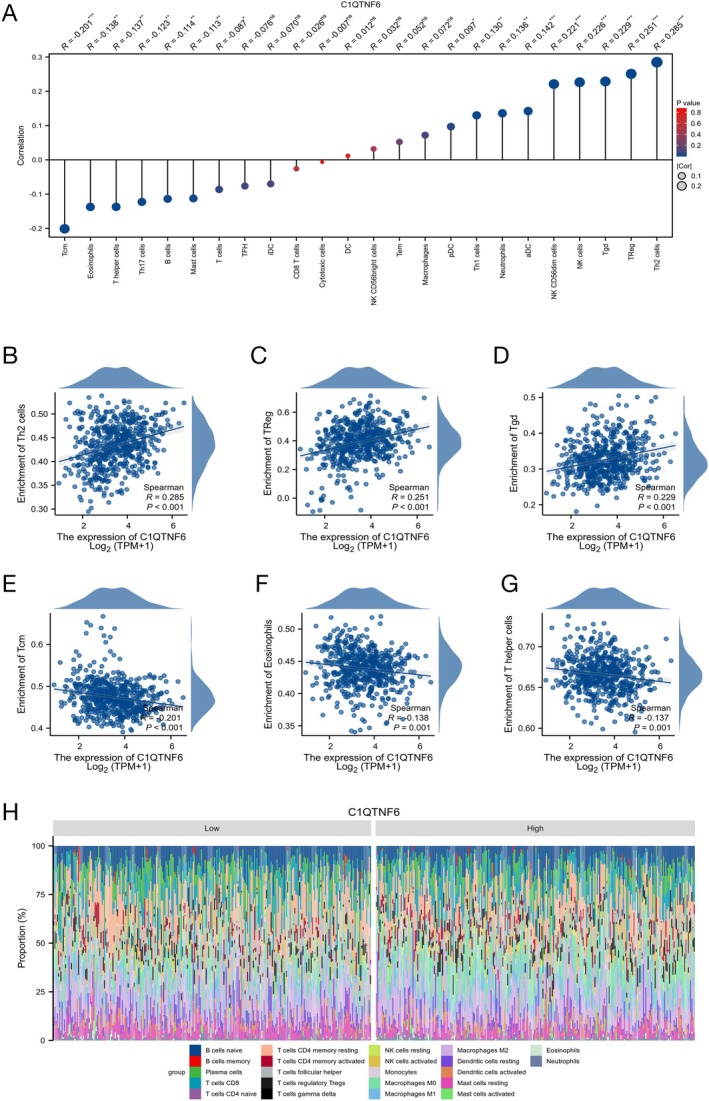
ssGSEA analysis of C1qtnf6 and its relationship with immune response. (A) Correlation analysis of C1qtnf6 expression and 24 genes in immune cell infiltration. (B–D) C1qtnf6 expression was positively correlated with Th2, TReg, and Tgd cells. (E–G) C1qtnf6 expression was negatively correlated with Tcm, eosinophils, and T helper cells. (H) Visualization of immune cells.

Tumor‐infiltrating lymphocytes are important for cancer development and affect the prognosis of patients with lung cancer. Thus, we investigated the possibility of a correlation between C1qtnf6 and the degree of immune cell infiltration in patients with LUAD. According to our findings, there is a substantial and positive correlation between Th2, TReg, and Tgd cells and the mRNA expression of C1qtnf6. Meanwhile, as shown in Figure 7A–C, a negative correlation was observed between Tcm, Eosinophils, and T helper cells and C1qtnf6 expression. Using the difference in immune infiltration findings between the groups with high and low expression of C1qtnf6, we were able to observe immune cells (Figure 7D).

### 3.7 Correlation Analysis Examining the Association Between C1qtnf6 Expression and the Tumor Immune Microenvironment

**FIGURE 8:**
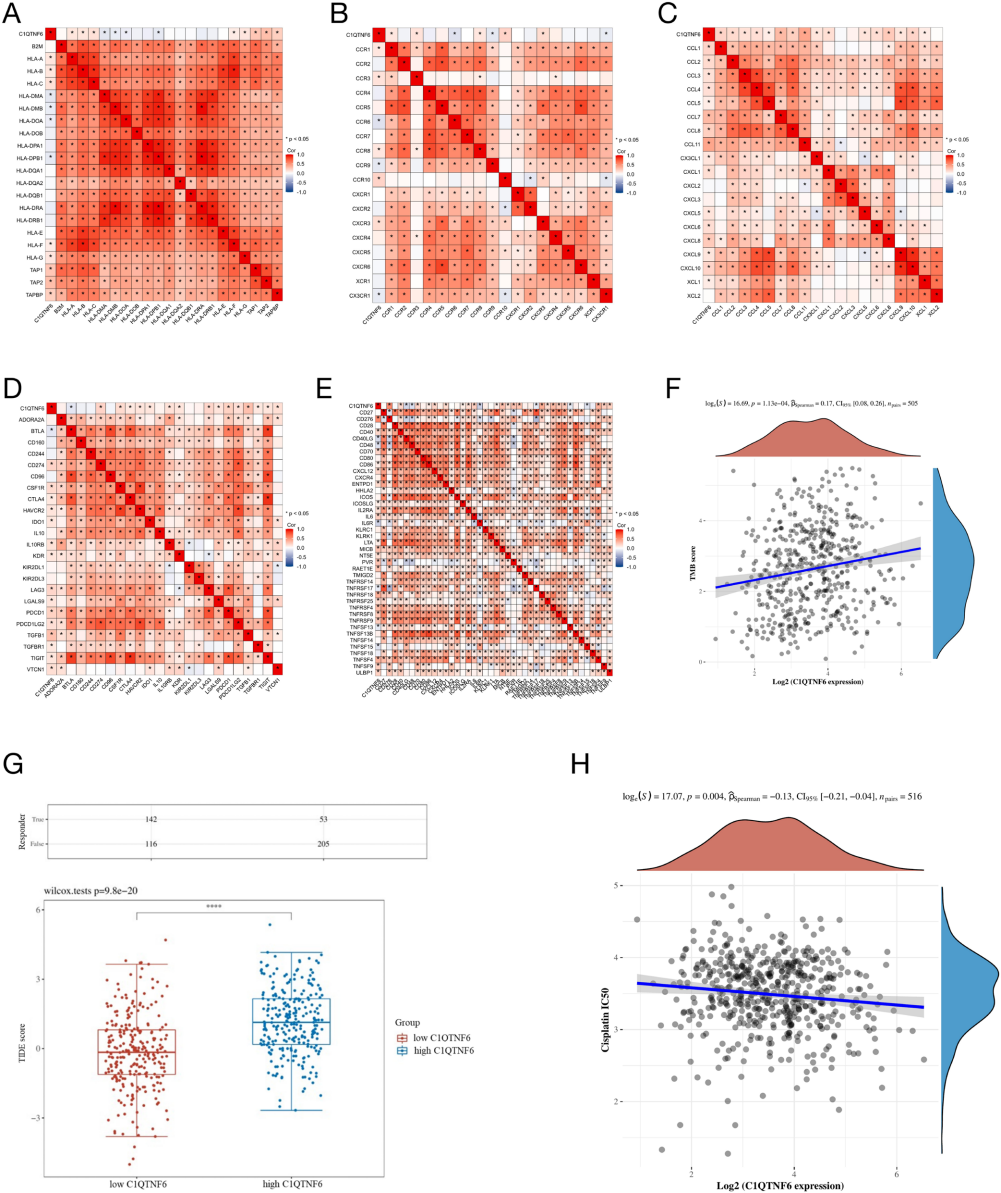
Correlation analysis of C1qtnf6 expression and tumor immune microenvironment. Analysis of *C1qtnf6* coexpression with MHC genes (A), chemokine receptors (B), chemokine genes (C), immunostimulatory factors (D), and immunosuppressor factors (E). (F) C1qtnf6 expression and TMB score. (H) In LUAD patients, the C1qtnf6 group showed a response different from that of immune checkpoint suppression. (I) C1qtnf6 expression and cisplatin IC_50_ dose.

We aimed to better understand the effects of C1qtnf6 on the immune system. We investigated this using immunosuppressants, chemokine receptors, immunological activators, and major histocompatibility complex (MHC) as coexpression molecules. Nearly all MHC marker genes (Figure 8A), chemokine receptors (Figure 8B), chemokine genes (Figure 8C), immunosuppressants (Figure 8E), and immunostimulators (Figure 8D) were coexpressed with the C1QTNF6 receptor. Additionally, patients with glioma with higher C1qtnf6 expression demonstrated higher TIDE scores, suggesting a potentially beneficial response to immune checkpoint blockade (ICB) treatment (Figure 8G). C1qtnf6 expression was favorably correlated with tumor mutational burden (TMB) scores (Figure 8F). Furthermore, our investigation of C1qtnf6 revealed a negative correlation with cisplatin, a widely used drug (Figure 8H).

### 3.8 Pathway Analysis and Gene Function Annotation of C1qtnf6

**FIGURE 9:**
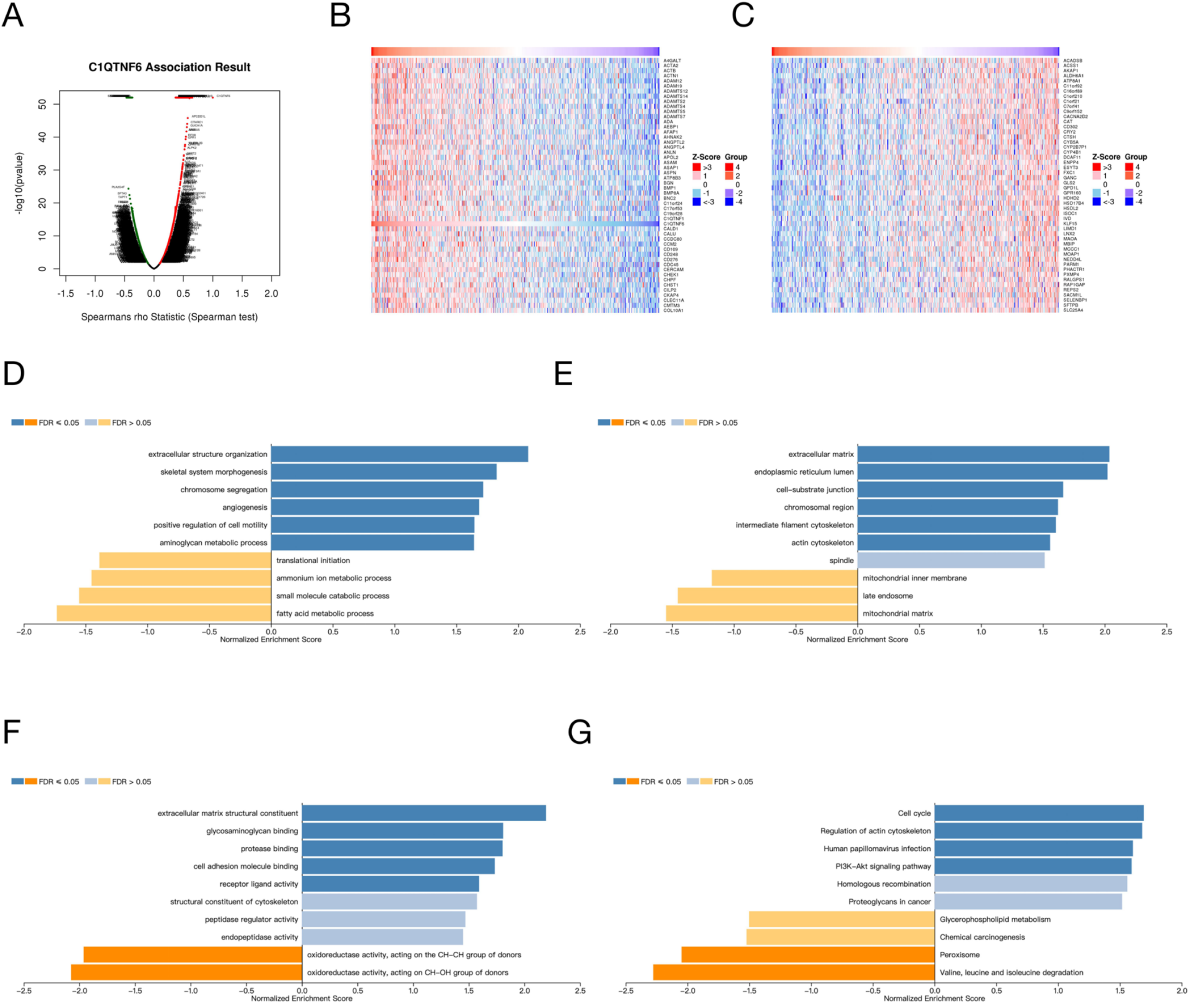
(A) Volcano maps of differentially expressed genes; (B, C) Heatmaps of differentially expressed genes. (D–G) GO and KEGG enrichment analysis of differentially expressed genes.

Significant positive correlations of C1qtnf6 were obtained using LinkedOmics, and the heat map displayed 50 positive associations (Figure 9A–C). GO analysis and KEGG enrichment were utilized to examine the functions of *C1qtnf6* among several differentially expressed genes to investigate the possible involvement of C1qtnf6 in LUAD. Changes in the biological processes, cell components, and molecular functions of C1qtnf6 were correlated with extracellular structure organization, extracellular matrix interactions, and oxidoreductase activity (Figure 9D–F). KEGG analysis showed that C1qtnf6 was also involved in the molecular pathway of the cell cycle (Figure 9G).

### 3.9 *C1qtnf6* Knockdown Inhibits Cell Proliferation

**FIGURE 10:**
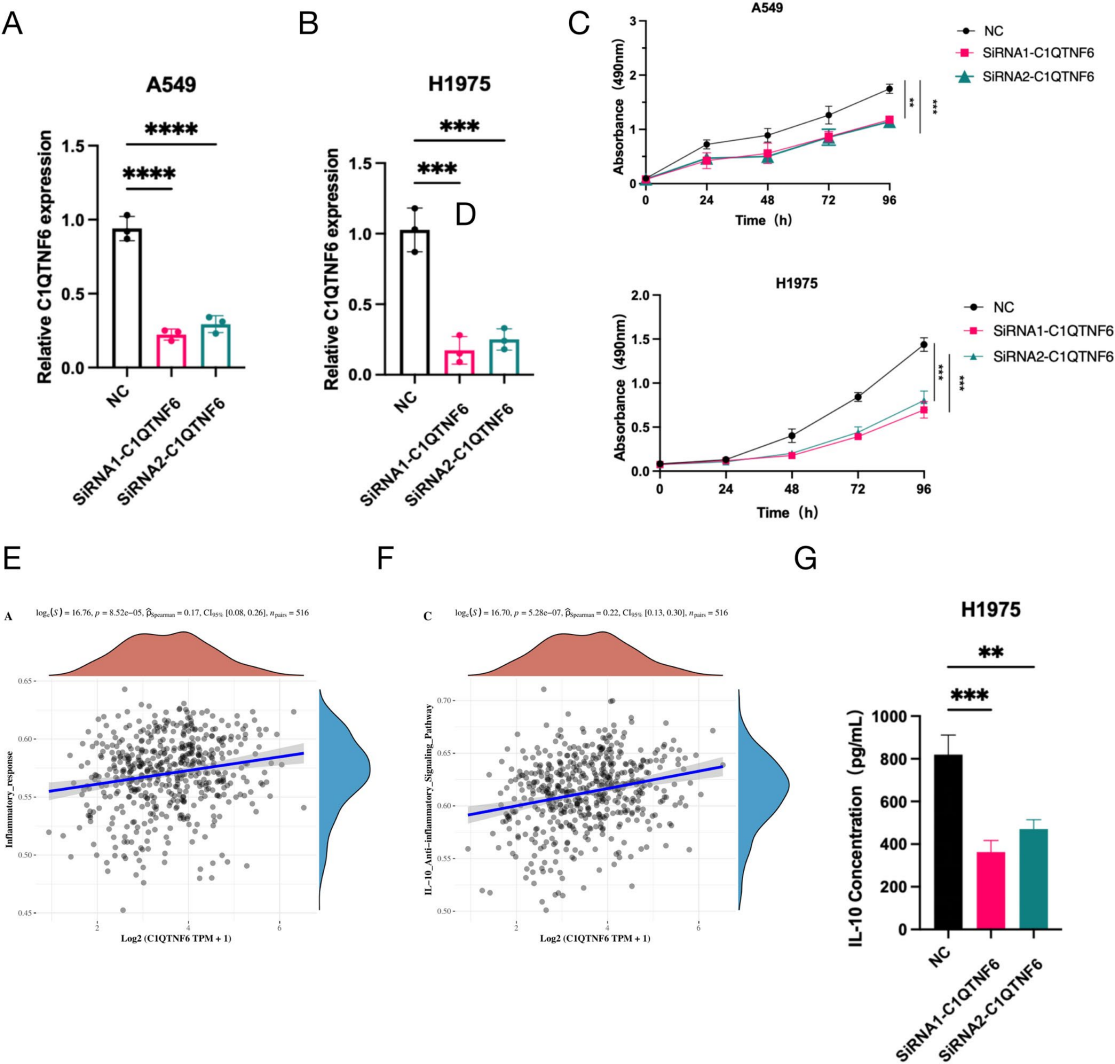
(A, B) Downregulation of C1qtnf6 expression in A549 and H1975 cells treated with siRNA, compared to control cells. (C, D) MTS assay showing the inhibitory effect of siRNA‐mediated *C1qtnf6* knockdown on the proliferation of A549 and H1975 cells over time. (E) Correlation between C1qtnf6 expression and inflammatory response markers. (F) Correlation between C1qtnf6 expression and IL‐10 levels. (G) Changes in IL‐10 levels following *C1qtnf6* knockdown in H1975 cells.

Two siRNAs were used to inhibit *C1qtnf6* expression in A549 and H1975 cells. To determine how well the siRNAs interfered, RT‐qPCR was performed following transfection and a 48‐h incubation period. The higher silencing effectiveness was achieved using the siRNA (Figure 10A,B). MTS experiments comparing the *C1qtnf6* gene knockouts NC group revealed that the proliferation of A549 and H1975 cells was reduced compared to that in the NC group (Figure 10C,D). The scatter plot shows that C1qtnf6 was positively correlated with the inflammatory response (Figure 10E) and IL‐10 levels (Figure 10F). ELISA showed that IL‐10 levels decreased after silencing *C1qtnf6* in H1975 cells (Figure 10G).

### 3.10 *C1qtnf6* Knockdown Promotes Apoptosis of Lung Cancer Cells

**FIGURE 11:**
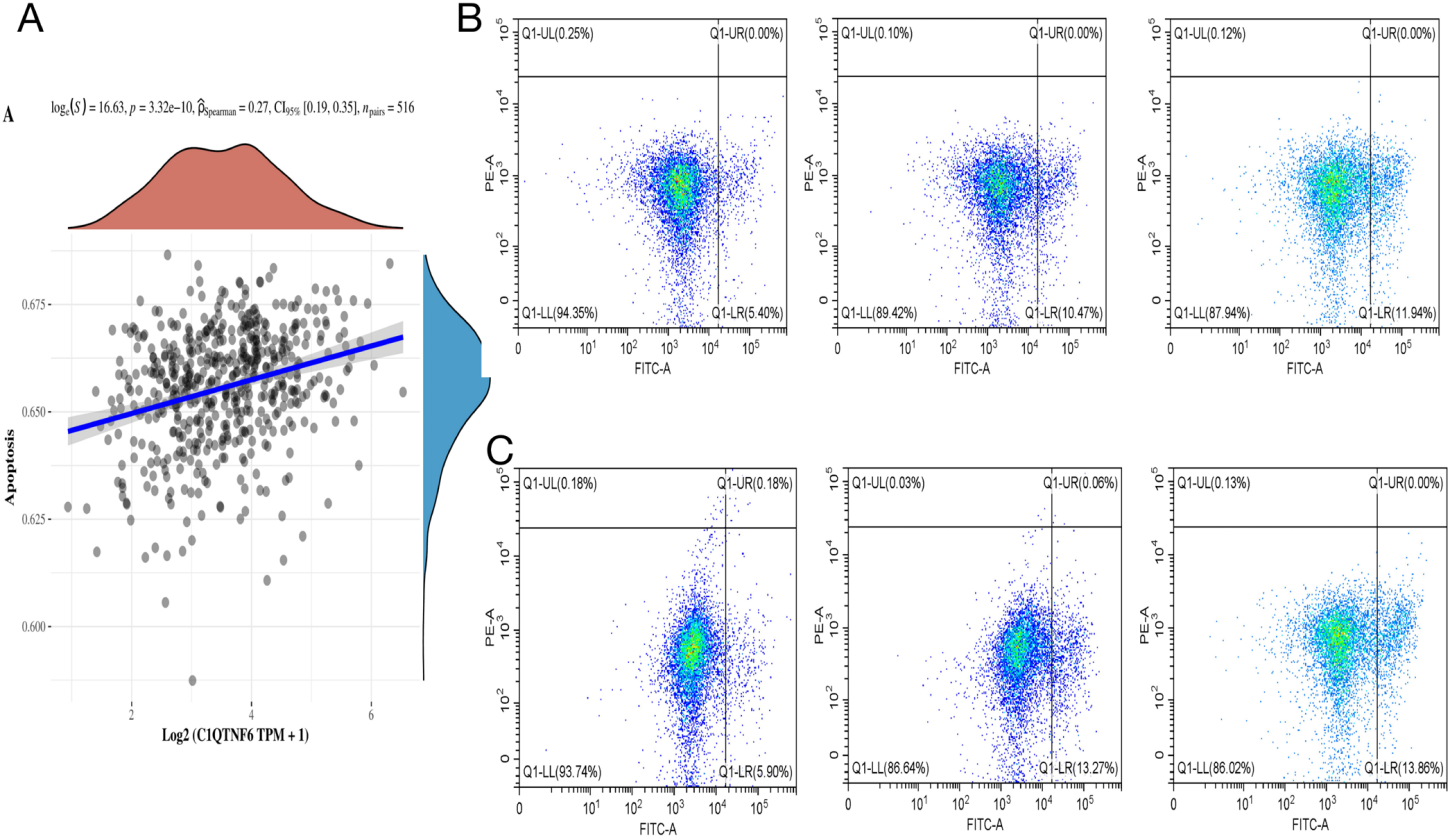
*C1qtnf6* depletion resulted in increased apoptosis. (A) Correlation between CIQTNF6 expression and apoptosis. (B, C) Flow cytometry of apoptosis under different conditions.

A correlation analysis revealed a strong relationship between C1qtnf6 expression and apoptosis (Figure 11A). The extent of apoptosis in lung cancer cells was assessed using FITC/PI double staining. Following *C1qtnf6* knockdown, increased apoptosis was observed in both H1975 and A549 lung cancer cells. Compared with the control group, the rate of apoptosis in A549 cells increased from 5.40% to 11.94%, whereas that in H1975 cells increased from 6.08% to approximately 13.86% (Figure 11B,C).

## 4 Discussion

In this study, we examined the mRNA expression of *C1qtnf6* in pan‐tumor tissues and the equivalent nearby normal tissues using TCGA and GTEX datasets [22]. This study revealed that C1qtnf6 is consistently upregulated in several human cancers, which is consistent with previous analyses. In terms of predictive and prognostic value, the ROC values for C1qtnf6 in LUAD demonstrated high sensitivity and specificity, comparable to or exceeding those of several established lung cancer biomarkers. Furthermore, although C1qtnf6 expression is elevated in various cancer types, its unique pattern of elevated expression in LUAD suggests that it may offer specific prognostic insights in this cancer type. However, to fully assess its specificity, future studies should investigate whether C1qtnf6 expression is also elevated in nonmalignant lung diseases, such as COPD or pulmonary fibrosis, to determine its potential diagnostic boundaries. We observed a notable increase in both C1qtnf6 RNA and protein expression, specifically in LUAD tissues, which correlated with unfavorable clinical characteristics including pathological and TNM stages and OS events. Remarkably, our ROC curve analysis indicated that C1qtnf6 holds promise as a diagnostic biomarker for distinguishing LUAD from normal tissues. Given this finding, it is worth exploring whether C1qtnf6 expression could also serve as an early detection marker. Further studies should assess its sensitivity and specificity for identifying early‐stage LUAD compared to noncancerous tissues, potentially enhancing early diagnostic strategies. Additionally, among patients with LUAD in the TCGA dataset, the Kaplan–Meier curve showed a link between C1qtnf6 expression and OS, disease‐free survival, and PFS. These results demonstrate the role of C1qtnf6 in the development of lung cancer found in our study is similar to those of earlier studies that found high levels of C1qtnf6 in various human cancer tissues, coupled with related clinicopathological characteristics and poor prognosis.

Enhanced *C1qtnf6* expression in LUAD is associated with a poor prognosis and decreased survival. This study is among the first to reveal a relationship between high C1qtnf6 expression in LUAD and specific immune infiltration patterns. This discovery expands our understanding of the role of C1qtnf6 in the tumor microenvironment and provides new evidence for its potential as an immune‐targeted therapeutic candidate for LUAD. Additionally, the role of C1qtnf6 as a prognostic marker is clinically significant, as it may help identify patients with poorer prognoses, supporting the development of personalized treatment strategies. According to the analysis of the TCGA‐LUAD data [23], individuals with elevated C1qtnf6 levels experienced lower OS, DSS, and PFS. In univariate and multivariate Cox regression analyses [24], It was determined that C1qtnf6 is a distinct risk indicator for LUAD. Many cancer types overexpress C1qtnf6, and many malignancies have C1qtnf6 linked to numerous traditional pathways, including epithelial–mesenchymal transformation (EMT) [25] and angiogenesis [26]. Based on these results, C1qtnf6 could be a viable target for cancer treatment and could aid in the study of the processes underlying anticancer drug resistance.

Immune cell infiltration is a common phenomenon observed in many cancer types. When cancer cells develop, they stimulate the immune system to respond by attracting various immune cells to infiltrate the tumor microenvironment. Immune cells, including macrophages, dendritic cells, T and B cells, and natural killer (NK) cells, are involved in the intricate interaction between the tumor and the immune system, which is crucial for the development and recurrence of cancer. C1qtnf6 is closely associated with immune cell infiltration, as seen from ssGSEA. C1qtnf6 and Th2 cells are positively correlated, whereas C1qtnf6 and Tcm are negatively correlated.

However, the precise functions and regulatory mechanisms of C1qtnf6 remain unclear. Therefore, this study aimed to shed light on these aspects. Through an analysis of lung cancer cases in the TCGA database, we discovered that C1qtnf6 exhibits high expression in the tumor tissues of lung cancer patients, which correlates with poorer OS rates. These results imply that C1qtnf6 contributes to lung cancer by acting as an oncogene. Additionally, MTS and flow cytometric analyses revealed that lung cancer cell growth and apoptosis are both enhanced by C1qtnf6. Future studies should delve deeper into the specific regulatory mechanisms associated with C1qtnf6 in patients with LUAD. Further investigating C1qtnf6 in the context of LUAD would establish its potential as a therapeutic target in the clinical treatment of lung cancer; hence, compilation of further evidence is warranted.

Our study has several limitations. First, all the analyses relied on online databases, which means that our conclusions would greatly benefit from further confirmation through clinical studies. Second, to comprehend the mechanism of C1qtnf6‐mediated immune infiltration in lung cancer more thoroughly, conducting both in vitro and in vivo studies is crucial. These investigations would advance the knowledge of the C1qtnf6 function in the immune infiltration mechanism in lung cancer and offer insights into the specifics.

## Author Contributions

**Qincai Li:** investigation, visualization, writing – original draft, writing – review and editing. **Hua Zhang:** investigation. **Hai Yun‐Liu:** formal analysis. **Peifeng Zheng:** supervision. **Xiaogang Xu:** investigation, funding acquisition, visualization, supervision.

## Conflicts of Interest

The authors declare no conflicts of interest.

## Data Availability

The data that support the findings of this study are available on request from the corresponding author. The data are not publicly available due to privacy or ethical restrictions.
